# Environmental, land cover and land use constraints on the distributional patterns of anurans: *Leptodacylus* species (Anura, Leptodactylidae) from Dry Chaco

**DOI:** 10.7717/peerj.2605

**Published:** 2016-11-03

**Authors:** Regina Gabriela Medina, Maria Laura Ponssa, Ezequiel Aráoz

**Affiliations:** 1 Unidad Ejecutora Lillo (UEL), CONICET-Fundación Miguel Lillo, San Miguel de Tucumán, Tucumán, Argentina; 2 IER (Instituto de Ecología Regional), Universidad Nacional de Tucumán, Yerba Buena, Tucuman, Argentina

**Keywords:** Conservation, Chaco, Anura, Ecological Niche Model, Land cover, *Leptodactylus*, Distribution

## Abstract

Subtropical dry forests are among the most vulnerable biomes to land transformation at a global scale. Among them, the Dry Chaco suffers an accelerated change due to agriculture expansion and intensification. The Dry Chaco ecoregion is characterized by high levels of endemisms and species diversity, which are the result of a variety of climates and reliefs, allowing a wide variety of environments. The amphibian group exhibits a high richness in the Dry Chaco, which has been barely studied in relation to land cover changes. We used ecological niche models (ENMs) to assess the potential geographic distribution of 10 *Leptodactylus* species (Anura, Leptodactylidae), which are mainly distributed within the Dry Chaco. We characterized these distributions environmentally, analyzed their overlap with land cover classes, and assessed their diversity of ecoregions. Also, we evaluated how these species potential distribution is affected by the transformation of land, and quantified the proportional area of the potential distribution in protected areas. We found that temperature seasonality is the main constraint to the occurrence of the species studied, whose main habitats are savannas, grasslands and croplands. The main threats to these species are the effects of climate change over spatial patterns of seasonality, which could affect their breeding and reproduction mode; the loss of their natural habitat; the exposure to contaminants used by intensive agriculture and their underrepresentation in protected areas.

## Introduction

Habitat destruction and fragmentation produced by changes in land use and land cover (LULC), and climate change are major factors influencing the global decline of populations and species (Bennett & Saunders, 2010). The humid tropics and amazon basin were the main focus of research and debate in relation to the effects of land transformation over biodiversity (Aide et al., 2013). Nevertheless, in Latin America, dry forests and savanna/shrub biomes are experiencing the second highest rate of absolute deforestation, behind rainforests (Aide et al., 2013). The Great American Chaco–distributed across Argentina, Paraguay, Bolivia and small areas of Brazil–and particularly the Dry Chaco, is the only subtropical dry forest in the planet (*sensu* Olson et al., 2001). Since the early 1900s, the Dry Chaco has experienced extensive livestock ranching (Bucher & Huszar, 1999). At present, land use patterns are quite different across countries. The greatest annual rates of landscape transformation were registered in Paraguay, reaching 4% in 2010 (the highest historical values in the entire region), followed by Argentina (Vallejos et al., 2014). In Argentina, since the 1970s, the Dry Chaco suffered an accelerated change due to agriculture expansion and intensification, especially of soybean and implanted pastures (Zak, Cabido & Hodgson, 2004; Boletta et al., 2006; Grau, Gasparri & Aide, 2008). Both in Argentina and Paraguay, chacoan habitats destruction has been identified as one of the worst environmental disasters in South America (Taber, Navarro & Arribas, 1997; Vallejos et al., 2014). While deforestation for intensive agriculture is also occurring in Bolivia, most of the Bolivian Chaco is still forested (especially when compared to Argentina and Paraguay) (Taber, Navarro & Arribas, 1997). Today, the predominant natural vegetation of the Chaco corresponds to open woodlands of thorny forest, interspersed with grasslands (Morello et al., 2012). The subtropical Dry Chaco constitutes the second largest continuous forest, behind the Amazon rainforest (Eva et al., 2004) and it is the less fragmented dry forest ecosystem in the world (Portillo-Quintero & Sánchez-Azofeifa, 2010; Caldas et al., 2013). Consequently, it represents a major asset for continental-scale biodiversity conservation, which is highly threatened by LULC changes. Despite the current rate of habitat destruction and the ecosystem value of the Dry Chaco, the system of protected areas is scarce and inefficient in most of its extension, e.g. only 2% of the Argentinean Dry Chaco is protected under some type of legislation (Brown et al., 2006).

The Dry Chaco ecoregion is characterized by high levels of endemism and diversity of species, which are the result of a variety of climates and reliefs, deriving in a wide variety of environments (The Nature Conservancy et al., 2005). Thus, analyses assessing the impacts of LULC on the geographic patterns of distribution of taxonomic groups are of major importance, since different taxa are affected in different ways by land use and by its changes (Schulze et al., 2004; Leroux et al., 2010; Dallimer et al., 2012). Amphibians exhibit high richness in the Dry Chaco, which has been scarcely studied in association to land cover changes (Torres et al., 2014). This fact is worrying since amphibians have become a high-priority group for conservation efforts (de Pous et al., 2010; Urbina-Cardona & Flores-Villela, 2010; Trindade-Filho et al., 2012; Nori et al., 2013; Nori et al., 2015) due to the concern about declines in their populations, and amphibian species extinctions around the world (Young et al., 2001).

Anuran species of *Leptodactylus* inhabit both open (croplands, grasslands, and shrublands) and closed vegetation (forest) areas; thus, the environmental heterogeneity of the Dry Chaco can be explored through the distribution of the genus (de Sá et al., 2014). The genus is the most diverse in the Dry Chaco, representing 25% of the anuran species of the ecoregion (Cruz, Perotti & Fitzgerald, 1992; Brusquetti & Lavilla, 2006; Vaira et al., 2012), and at least one species of *Leptodactylus* has been declared as near threatened by the International Union for Conservation of Nature (2016). The genus shows the highest diversity of reproductive modes within Leptodactylidae family, the most diverse family of Neotropical anurans (200 spp). Conservation priorities of a certain taxonomic group may also inform about the conservation requirements of other groups (Rodrigues & Brooks, 2007). Under this assumption and taking into account that the most diverse group may be representative of a wide group of taxa, the genus *Leptodactylus* might be a good indicator to assess the responses of sensitive species to changes in LULC in the Dry Chaco ecoregion.

Current techniques of ecological niche models (ENMs) allow relating species distribution data (species occurrence at known locations) with information about the environmental and/or spatial characteristics of the locations (abiotic factors). The environmental conditions of the localities where a species occur provide of just a partial image of the niche which can be represented in the geographic space; thus, they may be informative about the potential distribution of the species (Lobo, 2015). This association leads to confusion between the concepts of Species Distribution Models (SDM) and ENM, since it is natural to talk about SDM when inferences from the occupied area are involved. However, if we try to model potential areas, which essentially involves geographic localities with favorable conditions for the occurrence of a species (i.e. conditions contained in its existing fundamental or realized niche, but which may be present in other unoccupied regions), we must use ENM concept (Peterson & Soberón, 2012). ENM is a robust method to characterize regional species distributions (Seoane et al., 2006; Ficetola et al., 2010), offering reliable information regarding environmental constraints. These spatial analyses are the basis for assessing the effects of LULC changes over the potential distribution of species, and for proposing conservation strategies.

In this study, we estimated the potential geographic distribution of 10 *Leptodactylus* species, which are mainly distributed within the Dry Chaco; and we characterized them environmentally, i.e. by assessing which environment variables are the most relevant to determine the occurrence of these species. To spatially characterize the distributions of *Leptodactylus* species, we analyzed the proportion of the ecoregions and the different land cover types in the full range of the potential geographic distributions. We used existing maps of cultivated areas from 1976 to 2013 to evaluate how the potential distributions of these species have been affected by the expansion of the agricultural frontier. To evaluate the state of protection of *Leptodactylus* chacoan species, we quantified the proportion of the potential distribution of these species included within protected areas, focusing on species included in International Union for Conservation of Nature (IUCN) conservation categories.

## Materials and Methods

### Selected species

We selected ten species of the genus *Leptodactylus*, whose main distribution area is in the Dry Chaco ecoregion: *Leptodactylus bufonius*, *L. chaquensis*, *L. elenae*, *L. fuscus*, *L. gracilis*, *L. laticeps*, *L. latinasus*, *L. latrans*, *L. mystacinus* and *L. podicipinus*. Among these species, *Leptodactylus laticeps* is the only one considered as near threatened by IUCN assessments (Cortez et al., 2004); and vulnerable by the categorization of Argentinean amphibians (Schaefer & Céspedez, 2012).

### Occurrence data

Presence points databases were constructed based on revised specimens of collections, databases of herpetological collections, bibliography and free databases. The material revised proceeded from the following herpetological collections: Argentina: Fundación Miguel Lillo (FML), Museo Argentino de Ciencias Naturales (MACN), Museo de la Plata (MLP), private collections of Dr. María Laura Ponssa (L) and Dr. Julián Lescano (JL); Brazil: Museu Nacional de Rio de Janeiro (MNRJ), Museu de Zoologia de São Paulo (MZUSP), and Paraguay: Instituto de Investigaciones Biológicas del Paraguay (IIBP), Museo Nacional de Historia Natural de Paraguay (MNHNP). The database of Laboratorio de Genética Evolutiva (LGE, Instituto de Biología Subtropical, Misiones, Argentina) was also considered in the analysis. One hundred thirty five scientific articles were analyzed to extract presence points. When presence points were scarce, we completed species records with data obtained from Global Biodiversity Facility Information GBIF (accessed in March 2016). We cleaned this information to avoid mistakes from outdated taxonomic arrangements. All presence records are available in Supplemental Information 1.

We confirmed the species identifications when it was possible to avoid primary source of error in niche modeling. We identified the species recognizing external morphology features proposed in literature as descriptions of species, taxonomic and phylogenetic revisions (e.g. Heyer, 1970; Heyer, 1978; Heyer, 2014; de Sá et al., 2014). We used a binocular stereoscope when it was necessary.

We used the geographic coordinates of each specimen to georeference species occurrences, using Google Earth whenever these coordinates were missing. We estimated the uncertainties of these locations by considering the extent of the locality: each locality was defined as a circle, with a point marking the most likely position (the geographic center of the named place), and a radius representing the maximum distance from the point within which the locality is expected to occur (Wieczorek, Guo & Hijmans, 2004). The database was cleaned of records which were outside the known altitudinal range of each species and whose uncertainty was higher than 10,000 m. Sampling bias in geographic space (i.e. localities which are overrepresented in the collections) may lead to biases in the inferences about the environmental requirements of the species. To avoid this spatial bias and its consequent autocorrelation, we disaggregated occurrences of each species at 10,000 m with ‘ecospat’ package (Broenniman et al., 2014; Boria et al., 2014) implemented in R software. The conserved occurrence points were plotted in maps to check the consistency with the known ranges of the species. We analyzed a total of 2,306 localities, and the number of occurrences per species varied between 33 for *L. laticeps* and 467 for *L. latrans* (Table 1).

**Table 1:** Compiled records: number of compiled records from different sources (herpetological collections, private anuran collections, bibliographic records and Global Biodiversity Information data base) with geographic coordinates. Unique records: number of unique locality records. Checked records: number of locality records whose specimens were checked from the total number of unique records. Records not checked: locality records whose specimens were not checked from the total number of unique records. Collections: not checked specimens of scientific collections. GBIF: locality records obtained from Global Biodiversity Information Facility, Bibliographic records: locality records obtained from literature. Disaggregate records: number of disaggregate records to 10,000 m.

Species	Compiled records	Unique records	Checked records	Records not checked			Disaggregate records
				Collections	GBIF	Bibliographic records	
*L. bufonius*	1,562	334	198	93	–	43	271
*L. chaquensis*	1,099	463	237	111	–	115	383
*L. elenae*	354	136	81	2	–	53	122
*L. fuscus*	1,562	1,056	476	83	303	194	772
*L. gracilis*	422	200	89	38	–	73	178
*L. laticeps*	93	43	30	11	–	2	33
*L. latinasus*	1,716	488	246	106	–	136	416
*L. latrans*	2,138	514	264	228	–	22	467
*L. mystacinus*	641	335	169	70	–	96	292
*L. podicipinus*	824	273	117	37	15	104	214
Total	10,411	3,842	1,907	779	318	838	3,148

### Ecological niche model and environmental characterization

To model the presence of a species, we used the theoretical Biotic-Abiotic-Mobility approach (BAM) of Soberon & Peterson (2005), which captures and links the geographic and environmental dimensions of species distributions (Peterson & Soberón, 2012). The BAM model is composed of three components: 1) “B” components are the biotic conditions, i.e. the appropriate suite of both present species (e.g. food) and certain absent species (e.g. strong competitors, diseases). In general, this component is not included in the modeling process due to the fact that it is very difficult to make accurate spatial quantification of this kind of data (Barve et al., 2011). According to this and considering that *Leptodactylus* species exhibit a broad food spectrum, for which considering the component B becomes much more difficult, we do not included it in the present work. 2) The “A” components represent the abiotic conditions, e.g. bioclimatic variables; and 3) The “M” component is the region of the world which has been accessible to the species via dispersal over relevant periods of time. This heuristic scheme assumes that stable populations of a species will be found only in the intersection of the B, A, and M components, (B∩A∩M) (Soberon & Peterson, 2005).

To delineate the “A” component, we selected environmental variables (i.e. climate and soil variables) matching the temporal and geographic resolution of the studied species occurrences. Climate variables refer to temperature and precipitation. Since anurans are ectotherms, they are more vulnerable to environmental changes than other tetrapods (Duellman & Trueb, 1994). Temperature is the most pervasive factor affecting the rate of biological reactions and physiological processes of amphibians (Rome, Don Stevens & John Alder, 1992); while precipitation is the most important extrinsic factor controlling the seasonality of reproduction in anurans (Duellman & Trueb, 1994). As a matter fact, in most of the species of temperate latitudes, reproductive activity depends on temperature and precipitation (Duellman & Trueb, 1994). Soil was hypothesized to be an important predictor of the distribution of arid-adapted anurans, since it can affect water loss rates during aestivation (Schalk, Montaña & Springer, 2015). Soil pH influences hatching success in *Pseudophryne bibronii* (Chambers, Wilson & Williamson, 2006), thus, soil characteristics could affect the success of the survival rate of terrestrial egg-laying species, such as most *Leptodactylus* species. Climate information was obtained from WorldClim database (Hijmans et al., 2005), which includes 19 variables of temperature and precipitation. Soil information was obtained from SoilGrids1km database (Hengl et al., 2014, through ISRIC–WDC Soils), which includes eight global soil variables that summarize different aspects of soil at six depths. Most of these variables are not independent; consequently, Pearson correlations were used to detect and exclude highly correlated variables (r ≥ 0.8). For soil variables, correlation analyses were made within each depth and between depths (2.5, 22.5 and 45 cm depth). When high correlations were detected, we kept the variables that most likely affect the ecology of anurans. Eight bioclimatic variables were retained: BIO1 = Annual Mean Temperature, BIO2 = Mean Diurnal Range (Mean of monthly (max temp − min temp)), BIO4 = Temperature Seasonality (standard deviation of mean month temperature × 100), BIO5 = Maximum Temperature of Warmest Month, BIO6 = Minimum Temperature of Coldest Month, BIO12 = Annual Precipitation, BIO13 = Precipitation of Wettest Month, BIO14 = Precipitation of Driest Month. Because the correlations of each soil variable between depths were high (r > 0.9), only the variables of the most superficial depth (2.5 cm) were kept. Six soils variables were retained: 1) Sand content, 2) Coarse Fragments, 3) Soil organic carbon, 4) pH index, 5) Bulk density, and 6) Cation-exchange capacity. The environmental databases used presented a spatial resolution of 2.5′ (approx. 5 × 5 km per pixel at the equator).

A critical step for calibration, validation and comparison of ENMs is the definition of the “M” component; i.e. the extent of the region where the model calibration will be performed. The “M” component must be customized for each species and must be related to an explicit a priori hypothesis (Barve et al., 2011). It is reasonable to take the set of biotic regions within which a species is known to occur as the hypothesis of the M region (Barve et al., 2011). Here, the M region was defined for each species by identifying the ecoregions (*sensu* Olson et al., 2001) with known presences and by buffering the resulting region to generate a soft edge. The buffer size was between 0.1° and 0.5°, depending on each species data. A buffer of 0.5° was applied when the points were located marginally in one ecoregion, and a smaller buffer (less than 0.5°), when the points were located more uniformly in the ecoregions. This prevents from including areas where the species had no access. We pruned the selected areas when they included some regions where species obviously did not occur. For example, since *Leptodactylus* species are mainly distributed in the lowlands, we excluded higher altitudes from the “M” regions according to the altitudinal range known for each species.

ENMs were calibrated using a maximum entropy method, MAXENT v3.3.3K (Phillips, Anderson & Schapire, 2006) in batch mode implemented in R-package “ENMGadgets” (Barve & Barve, 2016). Maxent fits models based on the probability distributions that show maximum entropy (i.e. closest to uniform), subject to the correlations of known presences of species with the environmental conditions across the study area (Phillips, Anderson & Schapire, 2006). Most default settings were kept but we defined five bootstrap replications and raw outputs (i.e. continuous probability values). We randomly partitioned occurrence data in two subsets: 50% of the occurrences were used to calibrate the model, and the remaining 50% were used for model evaluation. Given the observed limitations of the Receiver Operating Characteristics Curve (ROC) approach we used a partial ROC area under the curve (AUC) to evaluate the performance of the model (Lobo, Jiménez-Valverde & Real, 2008). Partial ROC allows for differential weighting of omission and commission errors and focuses on meaningful predictions for model evaluation (Peterson, Papeş & Soberón, 2008). We restricted ROC space to predictions corresponding to omission error ≤ 5%, and we then randomly sub-sampled 50% of the available evaluation data 1,000 times and estimated the ratios of simulated AUCs and null expectations of AUC (Peterson, Papeş & Soberón, 2008) using Partial-ROC software (Barve & Barve, 2016). Probabilities were determined by direct count of null replicate frequencies of AUC ratios falling below the observed value.

To identify the variables which contributed the most to the model, we used a jackknife test on the original environmental variables. This test removes one environmental variable from the full list at a time and recalculates the model to quantify the contribution of each variable to the overall model performance.

### Spatial analyses

The resulting ENM can be projected in the geographic space, thereby depicting the potential distribution area of each species (Araújo & Peterson, 2012). We assumed that the species analyzed are in ecological equilibrium (i.e. their populations are not migrating) and that the potential distribution obtained corresponds to their historical distribution. The assumption of equilibrium of a population within its ecological niche (or single environment) implies ignoring mutation pressures from the environment and migration processes (Levene, 1953). State of stable equilibrium should not be viewed as a primary property of ecological systems, but it is a feature that can emerge from extrapolation to large spatial scales (DeAngelis & Waterhouse, 1987). The estimates of the geographic range extent obtained by ENM techniques have proved to be more representative than those obtained by traditional methods (e.g. minimum convex polygon (MCP)), particularly when data are scarce or when species are rare (Marcer et al., 2013; Pena et al., 2014; Syfert et al., 2014); and also avoid potential subjective bias of experts (Fourcade et al., 2013). We used a minimum threshold (Anderson, Lew & Peterson, 2003) to convert raw model outputs (i.e. continuous probability values) to distribution estimates (i.e. a binary representation). We modified this threshold–which, by default, includes 100% of the training data–to consider the potential error in the occurrence data, by choosing the highest threshold which included (100–E)% of the training data, assuming E = 5% (Table 2). E is an estimate of the proportion of the occurrence data that is likely to include the georeferenciation error (Peterson, Papeş & Soberón, 2008). This is a conservative method that minimizes the commission rate. This threshold defines that 95% of the observed presences are predicted as such, representing, in other words, the sensitivity of the model (Allouche, Tsoar & Kadmon, 2006). We then projected the distributional estimates of the ENM in space to obtain a quantitative potential geographic distribution. The spatial reference used for the analyses was Lambert Azimuthal Equal Area (LAEA) projection at a spatial resolution of approximately 5 × 5 km per pixel at the equator. The analyses and maps were developed with QGis software v. 2.16.2 (QGIS Development Team, 2016).

**Table 2:** Threshold: conversion threshold values to convert raw model outputs (i.e. continuous probability values) to distribution estimates (i.e. a binary representation). Percentages of relative contributions of the environmental variables to each one species model.

Species	Threshold	Percentage of contributions of environmental variables													
		Annual mean temperature	Mean diurnal range	Temperature seasonality	Max temperature of warmest month	Min temperature of coldest month	Annual precipitation	Precipitation of wettest month	Precipitation of driest month	PH index	Bulk density	Cation exchange capacity	Soil organic carbon	Coarse fragments	Sand content
*L. bufonius*	0.20	5.91	2.19	10.82*	10.43*	1.17	5.54	6.07	10.07*	29.61*	2.92	2.75	6.23	3.43	2.85
*L. chaquensis*	0.12	8.10	1.67	43.34*	3.74	1.09	0.89	3.72	3.65	20.33*	2.11	4.11	0.69	1.35	5.21
*L. elenae*	0.12	3.70	4.44	34.50*	9.72	1.75	1.50	6.44	2.11	12.07*	3.66	11.57*	2.81	3.90	1.83
*L. fuscus*	0.19	5.08	3.89	19.69*	2.31	0.93	6.16	6.26	6.44	30.44*	1.47	9.48	2.84	4.44	0.57
*L. gracilis*	0.06	4.59	4.25	18.55*	5.16	2.16	3.99	2.82	3.08	12.15*	8.60	10.40*	17.22*	5.04	1.98
*L. laticeps*	0.21	2.37	0.85	5.56	33.63*	3.01	3.36	6.39	8.36	2.38	2.38	0.41	7.26	16.71*	7.32
*L. latinasus*	0.13	9.09	6.84	23.24*	2.36	4.62	8.77	9.85	3.41	6.86	1.90	12.03*	2.46	5.02	3.55
*L. latrans*	0.09	7.47	5.21	47.79*	1.00	1.23	6.21	4.12	3.19	7.63	2.96	9.24	1.25	1.03	1.67
*L. mystacinus*	0.12	5.51	2.43	24.05*	2.03	26.99*	3.46	8.54	3.77	7.39	6.19	2.85	2.31	2.42	2.06
*L. podicipinus*	0.11	2.97	3.10	18.96*	9.83	5.69	8.07	1.26	21.04*	16.59*	2.41	2.54	1.71	3.19	2.62

**Note:**

* Values show percentages of relative contributions of the environmental variables higher than 10%.

#### Land cover and ecoregion characterization

To spatially characterize the potential distribution of each *Leptodactylus* chacoan species, we quantified the proportion of their extent which overlapped with ecoregions (*sensu* Olson et al., 2001) and with the different categories of land cover for Latin America produced by Blanco et al. (2013). The 24 land cover categories (e.g. broadleaf forests, needle leaf forests, mixed forests, shrublands, grasslands, water bodies, urban areas and croplands) are based on the FAO/UNEP Land Cover Classification System (LCCS). We also assessed the relationship between *Leptodactylus* occurrences and major habitat types, by assigning land cover categories in two groups “Forest habitats” (e.g. Subtropical broadleaf deciduous and evergreen forests, Tropical broadleaf deciduous and evergreen forests) and “open habitats” (e.g. Croplands, Tropical, Subtropical and Temperate shrublands, Tropical, Subtropical and temperate grasslands).

#### Land transformation in the Dry Chaco

We assessed whether the potential distribution of *Leptodactylus* frogs matches that of chacoan cultivable areas, i.e. suitable agriculture areas which are likely to be transformed to either pastures or croplands. In order to evaluate this, we estimated the proportion of each species distribution area affected by agriculture expansion, taking into account cultivated plots of the entire Dry Chaco region from 1976 to 2013 (Vallejos et al., 2014).

#### Protected areas

To explore the degree of protection of *Leptodactylus* species, we quantified the proportional protected area within the full range of the potential geographic extent of each species, taking into account the protected areas categories I to VI assigned by the IUCN (Dudley, 2008) (Ia: Strict Nature Reserve; Ib: Wilderness Area; II: National Park; III: Natural Monument or Feature; IV: Habitat/Species Management Area; V: Protected Landscape/Seascape; VI: Protected area with sustainable use of natural resources) and National Parks, even those that were not included in any IUCN category. Shape files of the protected areas were obtained from the World Database of Protected Areas (IUCN & UNEP-WCMC, 2016).

## Results

### Ecological niche model and environmental characterization

The partial ROC tests indicated significant predictive ability of the models (*p* < 0.001). For all species, bioclimatic variables (temperature and precipitation) were the most important predictors. The temperature variables (temperature seasonality, the maximum temperature of warmest month and the minimum temperature of coldest month) were the most important variables for seven out of 10 species. Only for one species’ potential distribution (*L. podicipinus*) the precipitation (precipitation of wettest month) was important. For two species’ potential distribution a soil variable (PH index) was the most important (Table 2).

### Spatial analysis

**Figure 1:**
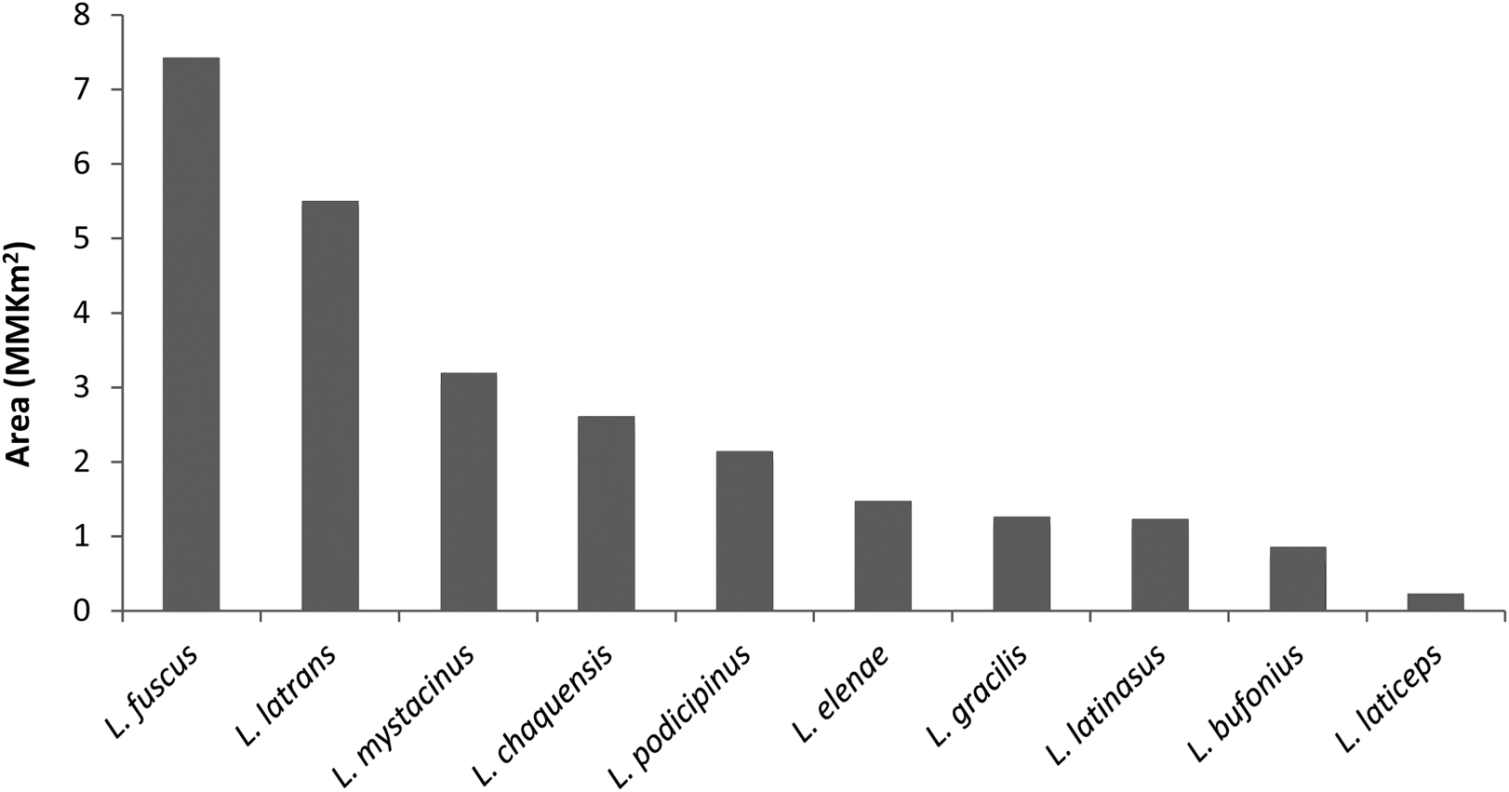
Distribution area (in MMKm^2^) of *Leptodactylus* chacoan species.

The potential distribution areas ranged from less than 40,000 km^2^ for *L. laticeps* to 7.5 million km^2^ for *L. fuscus*, with values between 1 and 3.5 million km^2^ for most species (Fig. 1). *Leptodactylus fuscus* and *L. latrans* were the species with the largest potential distribution ranges (above 5 million km^2^) (Fig. 1).

#### Land cover and ecoregion characterization

The level of overlaps of the potential distribution areas of *Leptodactylus* species ranged from 30 to 72% with “forest habitats” and from 29 to 71% with “open habitats” (Table 3). Among forest habitats categories, the highest overlaps were found in Subtropical broadleaf deciduous forests, Tropical broadleaf evergreen forests and Subtropical broadleaf evergreen forests. Among open habitat categories, the highest overlap occurred with croplands.

**Table 3:** Percentages of overlap of the distributions models of chacoan species of *Leptodactylus* with land cover categories from Blanco et al. (2013).

	Land cover	*L. bufonius*	*L. chaquensis*	*L. elenae*	*L. fuscus*	*L. gracilis*	*L. laticeps*	*L. latinasus*	*L. latrans*	*L. mystacinus*	*L. podicipinus*
Forest habitats	Tropical broadleaf evergreen forest	7.46	18.06*	28.21*	36.18*	0.36	1.93	0.30	21.12*	11.22*	34.70*
	Tropical broadleaf deciduous forest	0.55	0.27	0.47	0.15	0.00	0.06	0.00	0.08	0.09	0.29
	Sub-tropical broadleaf evergreen forest	5.87	8.56	9.79	6.37	23.38	0.68	6.65	9.34	11.28*	6.62
	Sub-tropical broadleaf deciduous forest	41.73*	14.95*	22.70*	5.03	5.00	69.12**	22.36*	6.73	10.99*	9.14
	Temperate broadleaf evergreen forest	0.00	0.00	0.00	0.01	0.03	0.00	0.01	0.03	0.02	0.00
	Temperate broadleaf deciduous forest	0.00	0.03	0.00	0.01	0.09	0.00	0.01	0.02	0.03	0.00
	Total	55.62**	41.87*	61.18**	47.74**	28.86**	71.80**	29.33**	37.32**	33.63**	50.75**
Open habitats	Tropical shrubland	0.09	6.70	6.86	15.09**	0.01	0.04	0.00	12.46**	7.70	9.55
	Tropical grassland	0.00	0.00	0.01	7.60	0.00	0.00	0.00	0.18	0.00	0.19
	Sub-tropical shrubland	17.32**	8.38	5.57	1.52	11.61**	10.07**	14.69**	4.69	7.28	4.61
	Sub-tropical grassland	0.30	1.80	0.01	0.36	10.29	0.00	10.61	2.60	3.80	0.36
	Temperate shrubland	0.00	0.11	0.00	0.00	0.83	0.00	0.03	1.90	1.17	0.00
	Temperate grassland	0.00	0.00	0.00	0.03	0.24	0.00	0.22	0.26	0.03	0.00
	Cropland	22.87**	36.25**	21.24**	24.99**	41.08**	15.17**	38.69**	36.27**	42.98**	29.24**
	Others	3.80	4.89	5.13	2.66	7.08	2.93	6.43	4.32	3.42	5.30
	Total	44.38**	58.13**	38.82**	52.26**	71.14**	28.20**	70.67**	62.68**	66.37**	49.25**

**Notes:**

* Values show overlap percentages higher than 10%.

** Values show overlap percentages higher than 50%.

**Figure 2:**
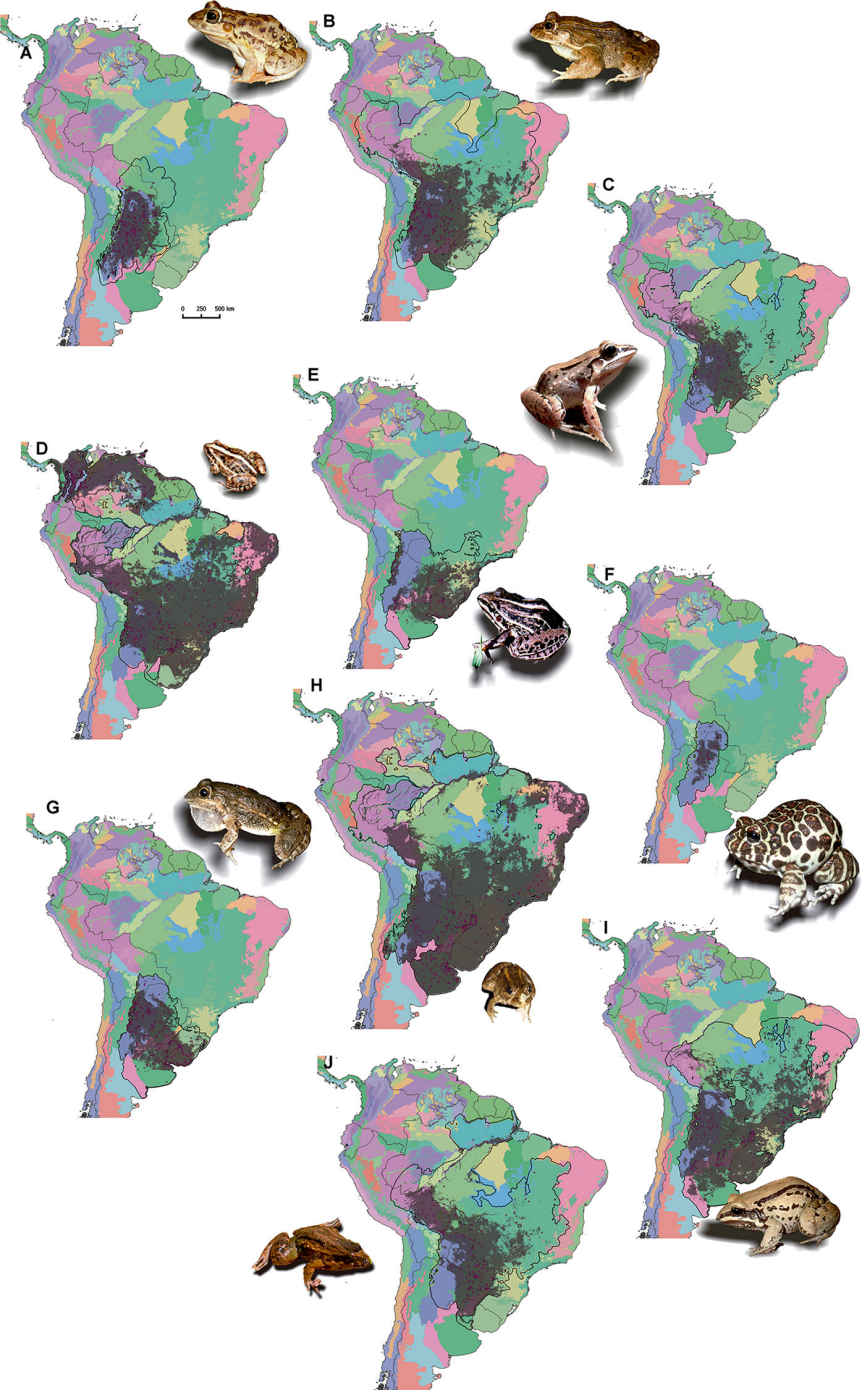
Maps for each *Leptodactylus* chacoan species, showing: distribution models, in grey; calibration area of the distribution model, (i.e. “M” region), in black line; presence data, in purple points. The different colors correspond to the ecoregions proposed by Olson et al. (2001); and illustrate that all the species considered are distributed beyond the Dry Chaco ecoregion. (A) *Leptodactylus bufonius* (B) *L. chaquensis* (C) *L. elenae* (D) *L. fuscus* (E) *L. gracilis* (F) *L. laticeps* (G) *L. latinasus* (H) *L. latrans* (I) *L. mystacinus* (J) *L. podicipinus*.

All the species considered in this study distributed beyond the Dry Chaco ecoregion (Fig. 2). *Leptodactylus laticeps*, *L. bufonius*, *L. latinasus, L. elenae* and *L. chaquensis* are mostly Dry chacoan species, with at least 25% of their geographic range occurring in the Dry Chaco. These species potentially inhabit less ecoregions than the other species (Supplemental Information 2). On the other hand, the remaining five species (*L. latrans*, *L. fuscus*, *L. podicipinus*, *L. mystacinus, L. gracilis*) potentially inhabit in a greater number of ecoregions (Supplemental Information 2).

#### Land transformation in Dry Chaco

**Figure 3:**
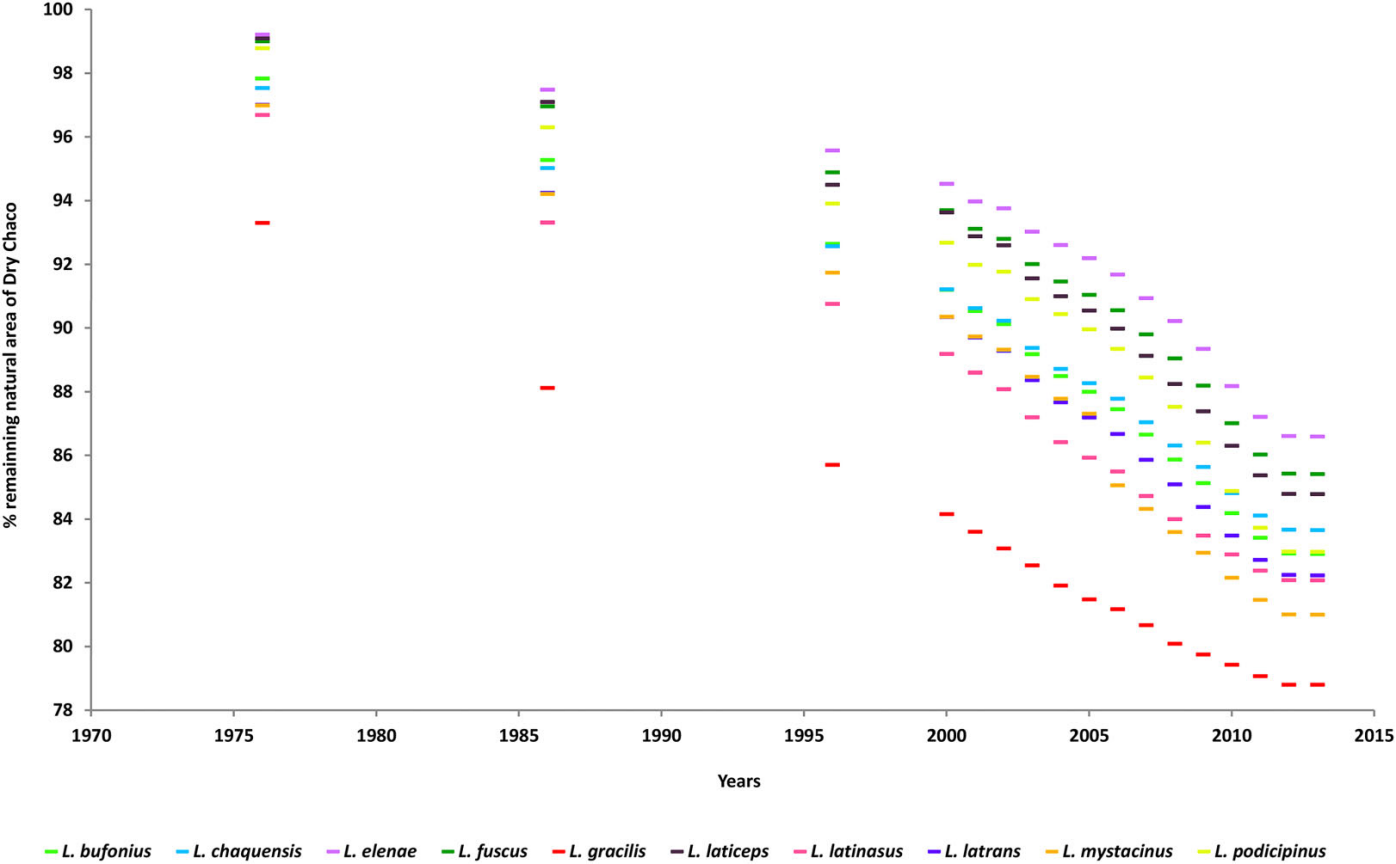
Percentage of transformation of natural area to cultivated plots in the Dry Chaco from 1976 to 2013, for each *Leptodactylus* chacoan species.

By the end of 2013, 1.8 million km^2^ of Dry Chaco (19% of the natural area in the entire ecoregion) were transformed to agriculture (Vallejos et al., 2014). The percentage of remaining natural areas inhabited by *Leptodactylus* chacoan species in the Dry Chaco decreased every year from 1976 to 2013 due to agricultural expansion, although the greatest transformation occurred after 1996 (Fig. 3). The largest percentage of potential chacoan area loss was 16% (*L. mystacinus* and *L. podicipinus*), while for seven species the loss of chacoan area were between 15% (*L. bufonius, L. gracilis, L. latinasus* and *L. latrans*) and 14% (*L. chaquensis, L. fuscus* and *L. laticeps*). The minimum loss of potential chacoan area was 13% (*L. elenae*) (Fig. 3).

#### Protected areas

**Figure 4:**
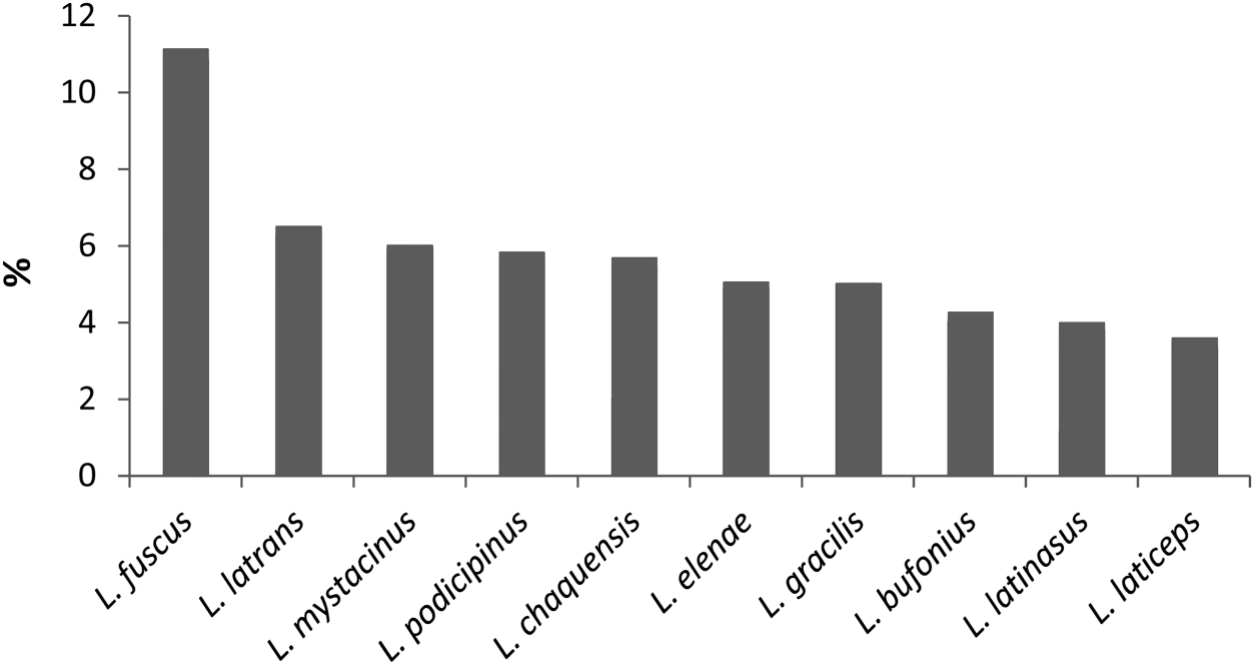
Percentage of protected area within the potential distribution range of *Leptodactylus* chacoan species.

**Figure 5:**
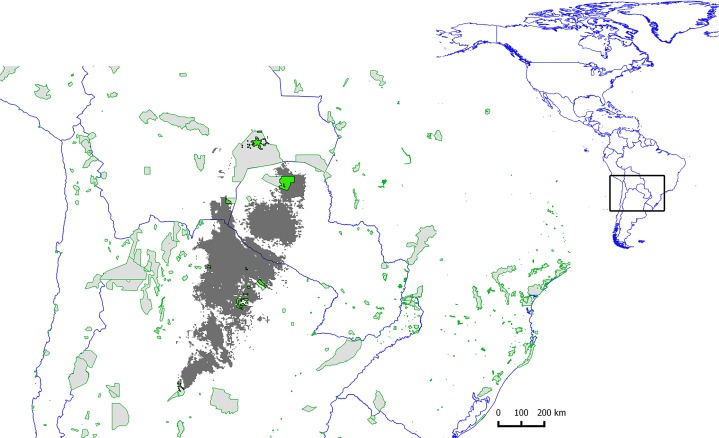
Map showing the potential distribution of *Leptodactylus laticeps*, in grey. Protected areas overlaying the potential distributions of *Leptodactylus laticeps* are filled polygons in green.

The highest proportion of the potential distribution area in protected areas was found in *Leptodactylus fuscus*, *L. latrans* and *L. mystacinus* (between 6 and 11%), while the remaining species (*L. bufonius*, *L. chaquensis, L. elenae*, *L. gracilis, L. latinasus, L. laticeps* and *L. podicipinus*) exhibited between 3.5 and 5% of their potential distribution in protected areas (Fig. 4). The near threatened (Cortez et al., 2004) and vulnerable (Vaira et al., 2012) species *Leptodactylus laticeps* showed only 3.6% of its potential distribution in protected areas (Fig. 5).

## Discussion

We found that the main constraining variable of the potential distribution of *Leptodactylus* chacoan species is temperature (temperature seasonality, maximum temperature of warmest month and minimum temperature of coldest month), and that these species are found in both forest and open areas. In the Dry Chaco ecoregion, they inhabit cultivable areas which are being transformed from natural vegetation to pastures and croplands. *Leptodactylus* species are underrepresented under the current system of protected areas. This combination of features suggests that *Leptodactylus* chacoan species are vulnerable to both climate change and habitat transformation. This situation is especially critical for *L. laticeps*, which besides being already threatened is distributed mainly in the Dry Chaco and is poorly represented in the system of protected areas.

### Ecological niche model and environmental characterization

Our results show that the most important bioclimatic variable explaining species occurrence (in 70% of the studied species) was temperature. Other studies have also shown that climate (especially temperature and rainfall) is a determinant factor for amphibians (e.g. Parris, 2004; Urbina-Cardona, Olivares-Pérez & Reynoso, 2006). Since amphibians are ectothermic organisms, environmental temperature is probably a major constraint to their ecophysiological traits; for example, their mobility and their energy balance may be affected by temperature (Bennett, 1990). Physiological traits turn amphibians strongly sensitive to environmental conditions (Ferder & Burrgren, 1992), and consequently to climatic change (Pounds, Fogden & Campbell, 1999; Pounds et al., 2006). Projected climate changes over the 21st century include rises in surface temperature, and in the frequency and duration of heat waves and the occurrence of extreme precipitation events (IPCC, 2014). Also, seasonality will be affected by more frequent hot temperature extremes and fewer cold temperature extremes over most land areas (IPCC, 2014). Particularly, in south-eastern South America, which includes Dry Chaco ecoregion, changes in precipitation produce wetter conditions (Christensen et al., 2007). Under these scenarios, it is expected that populations change their geographic ranges to coldest areas, i.e. to higher altitudes or latitudes (Harsch et al., 2009; Thomas, 2010; Chen et al., 2011), although in the case of amphibians, this is unlikely due to their low dispersal ability. Measurements of the home range size of amphibians show a mean home range size of 40 m^2^ (Wells, 2007). Moreover, the capacity for amphibian behavioral thermoregulation is limited because the cooling effect of evaporative water loss from skin counteracts heat gain by basking (Hutchinson & Dupe, 1992). Studies revealed that the effects of climate change over amphibians involve changes in the timing of breeding of some species (Blaustein et al., 2010; Carey & Alexander, 2003), instead of changes in their distributions (Corn, 2005). For example, no colonization of higher altitude areas associated with declines of *Anaxyrus boreas* has been found (Livo & Yeakley, 1997). In subtropical areas, the period of activity of amphibians and their population dynamics are strongly influenced by climate seasonality, e.g. reproduction behavior of most species occurs in the warm season of the year (Conte & Machado, 2005). For many species of *Leptodactylus* genus, the reproductive season matches with wet and/or warm season, e.g. *L. latinasus*, (Gallardo, 1964; Canavero et al., 2008), *L. bufonius* (Cei, 1980), *L. gracilis*, (Canavero et al., 2008; Ximenez & Tozetti, 2015), *L. mystacinus*, (Gallardo, 1964), *L. fuscus*, (Martins, 1988; Prado, Uetanabaro & Haddad, 2005; Lucas et al., 2008), *L. elenae* (Prado, Uetanabaro & Haddad, 2005), *L. chaquensis* (Gallardo, 1987; Prado, Uetanabaro & Haddad, 2005; Canavero et al., 2008) and *L. podicipinus* (Cei, 1980; Vizotto, 1967; Rossa-Feres & Jim, 1994); although it was reported that populations of *L. podicipinus* from the Brazilian pantanal exhibit a continuous reproductive cycle throughout the year (de Almeida Prado, Uetanabaro & Lopes, 2000; Prado, Uetanabaro & Haddad, 2005). The Dry Chaco presents a monsoonal climate, in which rains are concentrated in the warm season. Since the reproduction of most amphibians is associated to water, modifications in water balance in the area could have significant effects on their breeding timing and behavior. Thus, our results suggest that climate change could affect *Leptodactylus* chacoan species in different and unpredictable ways.

### Spatial analyses

#### Land cover and ecoregion characterization

In general, the *Leptodactylus* species studied inhabit both forest and open areas in similar proportions, with even a greater proportion in open areas. This result matches that of Torres et al. (2014), who found that the response of the probability of presence of all the analyzed amphibian species in Argentinean northwestern Dry Chaco to woody biomass was negative. The importance of open habitats for *Leptodactylus* species suggests that these species evolved in landscapes which were more similar to open habitats than to forests, a scenario proposed for the pre-European landscape of the Chaco (Morello & Saravia Toledo, 1959a; Morello & Saravia Toledo, 1959b; Adámoli et al., 1972; Adámoli et al., 1990; Bucher & Huszar, 1999), in which the substantial contemporary woodland advance has been a result of domestic livestock overgrazing (Morello & Saravia Toledo, 1959a; Morello & Saravia Toledo, 1959b; Adámoli et al., 1990). Thus, savannas might have been an important cover type where part of the chacoan biota evolved (Torres et al., 2014).

Among the open habitat categories, “croplands” was the most important for all species, which is also consistent with observational field data (e.g. Peltzer et al., 2006; Ponssa & Barrionuevo, 2008; Attademo et al., 2014; Prado & Rossa-Feres, 2014; Guerra & Aráoz, 2015). The IUCN categorization (2016) mentions that *L. bufonius*, *L. chaquensis*, *L. elenae*, *L. fuscus*, *L. latinasus, L. latrans*, *L. mystacinus* and *L. podicipinus* are or appear to be well adapted to anthropogenic disturbances, while *Leptodactylus gracilis* is found in anthropic areas only in southern Brazil (Heyer et al., 2004). The present study also shows that *L. gracilis* may inhabit anthropogenic areas, since 71% of their potential geographic range is covered by open habitats, from which 41% is covered by croplands. Moreover, *L. gracilis*, *L. latinasus* and *L. mystacinus* have been suggested to be invading species, due to their abundance in agricultural ponds, and their ability to constitute stable populations in these environments (Sanchez et al., 2013).

#### Land transformation in Dry Chaco

The dynamics of land cover transformation to agriculture shows that one half of analyzed *Leptodactylus* species may inhabit mainly cultivable areas. The percent loss of their potential distribution area within the Dry Chaco is near of the transformation rate of natural habitats to cultivated plots from 1976 to 2013. The largest percentages of decrease were found for *L. mystacinus* and *L. podicipinus*. This is particularly worrying for *L. laticeps* and *L. bufonius*, species which are distributed mainly in the Dry Chaco, which the loss of chacoan area represent the 14% and 10% of their entire potential distribution respectively. The entire dry diagonal region is suffering the same process of natural habitat loss described for the Dry Chaco, e.g. the Cerrado biome is being transformed for soybean production (Fearnside, 2001; Warnken, 1999). The Caatinga and the Cerrado (Beuchle et al., 2015), are also experiencing a continuous net loss of natural vegetation, and an expansion of sugarcane crops in the ‘‘dry diagonal’’ (Manzatto et al., 2009). This scenario represents potential threats to species with high percent loss in the Dry Chaco (mentioned above) and to those inhabiting the “Dry Diagonal,” such as *L. latrans* and *L. podicipinus*. The projected increase in annual rainfall may accelerate deforestation rates due to agriculture expansion, which would have a significant effect in the conservation of *Leptodactylus* chacoan species. It has been observed that deforestation in the Chaco is to some extent controlled by annual rainfall (Grau, Gasparri & Aide, 2005). This may be especially relevant for the conservation of *L. laticeps.* At least 20% of the potential distribution area of this species, which is already threatened, has already been transformed to agriculture in the last 40 years. Additionally to habitat loss and/or fragmentation in the Dry Chaco, the fact that *Leptodactylus* anurans potential geographic range currently corresponds to croplands and cultivable areas raises concern about the potential threats to which they may be exposed, e.g. contamination. It has been shown that anuran species breeding within or around agricultural areas are usually exposed to pesticides (Peltzer, Lajmanovich & Beltzer, 2003; Peltzer et al., 2006), which produce teratogenic effects, growth and development retardation, and consequently, decreasing survivorship (Hayes et al., 2006). This could be a serious risk for these well adapted to anthropogenic disturbances *Leptodactylus* species, and particularly for *Leptodactylus latinasus, L. chaquensis, L. latrans, L. mystacinus, L. gracilis* and *L. fuscus*, whose reproduction habits involve the exploitation of soil depressions in cultivated areas (Vasconcelos & Rossa-Feres, 2008; Silva & Rossa-Feres, 2007; Guerra & Aráoz, 2015; Peltzer et al., 2006).

Most of the anuran species undergo ontogenetic niche shifts (aquatic larvae and terrestrial adults); which is the case of *Leptodactylus* anurans, whose clutch deposition mode involves aquatic or terrestrial foam nests, aquatic larvae, and terrestrial adults. Thus, anthropic landscape transformation may produce a “habitat-split” (Becker et al., 2007), i.e. a spatial separation between remnants of terrestrial habitat and breeding sites (Dunning, Danielson & Pulliam, 1992). Habitat split presents a strong negative effect on anuran species with aquatic larvae, and is a determinant factor of population size, structure, and distribution, acting within a single generation (Becker et al., 2007).

#### Protected areas

We found that the percentages of *Leptodactylus* species distributions represented in protected areas are scarce (11% or less). Consequently, most of their geographical ranges (i.e. approximately 90%) may be vulnerable to human disturbance. A similar pattern was found for threatened anurans of Northeastern Brazil (*Adelophryne baturitensis, Adelophryne maranguapensis, Allobates olfersioides* and *Agalychnis granulosa*) (Campos, Brito & Solé, 2013). Added to the underrepresentation of chacoan species of *Leptodactylus* in protected areas, it has been found that the current assignment of protected areas to IUCN categories does not correspond to the expected gradient of naturalness (Leroux et al., 2010; Bishop et al., 2004; Chape et al., 2005; Dudley, 2008). Leroux et al. (2010) showed that the global protected areas network lacks of strictly-protected areas with low human influence.

*Leptodactylus laticeps* is under Near Threatened and Vulnerable conservation categories by Cortez et al. (2004) and the Argentinian assessment (Vaira et al., 2012), respectively. Our results show that *L. laticeps* inhabits the Dry Chaco ecoregion almost exclusively, mainly the Subtropical broadleaf deciduous forest (a typical natural land cover of Dry Chaco). The land transformation analysis showed that this species inhabits cultivable lands inside the Dry Chaco ecoregion, which, in combination with the underrepresentation of *L. laticeps* in protected areas and the expansion of agriculture around chacoan protected areas, increases the risk of ecological isolation (Matteucci & Camino, 2012). In Copo National Park, an Argentinean protected area of Dry Chaco, the increase in human corridors (e.g. roads) between 1976 and 1988 has been followed by an increase of the surrounding parceled land between 1988 and 2007 (Matteucci & Camino, 2012). Furthermore, habitat connectivity is severely compromised in the Argentinean Dry Chaco, where there are no ecological corridors connecting protected areas (Burkart, 2007). In Paraguay, actions to keep habitat connections have been limited to proposals such as the Biodiversity Corridor of the Dry Chaco (Comisión Mundial de Áreas Protegidas Paraguay, 2007). This proposal includes protected areas located at the northern end of the potential distribution range of *L. laticeps*: Teniente Agripino Enciso, Médanos del Chaco and Defensores del Chaco National Parks, the Natural Monument Cerro Timane Cabrera, Guasú Natural Reserve; and the Kaa Illa National Park in Bolivia. Also, the protected area systems in Latin America are deficient in the administration of economic resources, equipment, human resources and legal and regulatory frameworks (Brown et al., 2006; Castaño-Uribe, 2008). For instance, in Latin America and the Caribbean region: 1) Paraguay presents the lowest number of human resources per unit of protected area and the lowest rate of investment resources of the nation by protected hectare; 2) in Argentina, the staff has no specialized functions, lacking of own equipment; and 3) from 2001 to 2006, both in Argentina and in Paraguay, the amount of money allocated to protected areas decreased (Castaño-Uribe, 2008). In addition to these global concerns, it has been reported that *L. laticeps* species suffer of international commercial exploitation for pet trade (Schaefer & Céspedez, 2012; Cortez et al., 2004).

## Conclusions

To conclude, the main constraint to the potential distribution range of *Leptodactylus* chacoan species is temperature. Consequently, climate change associated to modifications of seasonality patterns could affect the breeding time and reproductive mode of these anurans. Land transformation for agriculture activities in the Dry Chaco exhibits an increasing trend in natural habitats rate of loss, where *Leptodactylus* chacoan species inhabit. This implies three potential adverse effects on amphibians: 1) habitat loss; 2) “habitat split,” produced by landscape fragmentation; and 3) exposure to contaminants used by intensive agriculture activities. The state of *Leptodactylus* protection is deficient, due to the scarcity of protected natural habitats in the region, and the underrepresentation of the potential distribution range of *Leptodactylus* species within protected areas (on average, around 5% of their potential distributions). This situation implies a strong concern about *L. laticeps* populations, whose main threats are agriculture activities and hunting pressure. In the Dry Chaco, open areas, such as savannas, grasslands and croplands, are the main habitat of *Leptodactylus* species. However, the current conservation focus in the Dry Chaco is on forest habitats, for which conservation planning should be redesigned to take into account the natural savannas and grasslands, in order to more comprehensively protect and account for habitat heterogeneity.

## Supplemental Information

10.7717/peerj.2605/supp-1 Supplemental Information 1 Locality data information of all unique records compiled for *Leptodactylus bufonius*, *L. chaquensis*, *L. elenae*, *L. fuscus*, *L. gracilis*, *L. laticeps*, *L. latinasus*, *L. latrans*, *L. mystacinus* and [i]*L. podi*. Locality data information includes collection number whenever available, museum acronym following Sabaj Perez (2010), locality data description, latitude and longitude in decimal degrees, and the bibliographical reference from which the information was obtained.

10.7717/peerj.2605/supp-2 Supplemental Information 2 Percentages of potential distribution of *Leptodactylus spp*. Percentages of potential distribution of *Leptodactylus bufonius*, *L. chaquensis*, *L. elenae*, *L. fuscus*, *L. gracilis*, *L. laticeps*, *L. latinasus*, *L. latrans*, *L. mystacinus* and *L podicipinus* on each ecoregion (sensu Olson et al., 2001). Values with asterisk show overlap percentages higher than 10%.

## Additional Information and Declarations

### Data Deposition

The following information was supplied regarding data availability: The raw data are included in the manuscript and as Supplemental Dataset Files.
