# Supplementary material for: Environmental, land cover and land use constraints on the distributional patterns of anurans: Leptodacylus species (Anura, Leptodactylidae) from Dry Chaco
Source: PeerJ. 2016 Nov 3;4:e2605. doi: 10.7717/peerj.2605 (PMC5101610; doi:10.7717/peerj.2605)
Supplement: Supplemental Information 2 — Percentages of potential distribution of Leptodactylus bufonius, L. chaquensis, L. elenae, L. fuscus, L. gracilis, L. laticeps, L. latinasus, L. latrans, L. mystacinus and L podicipinus on each ecoregion (sensu Olson et al., 2001). Values with asterisk show overlap percentages higher than 10%. [file peerj-04-2605-s002.docx]

Supplementary Material S2

|  | ***L. bufonius*** | ***L. chaquensis*** | ***L. elenae*** | ***L. fuscus*** | ***L. gracilis*** | ***L. laticeps*** | ***L. latinasus*** | ***L. latrans*** | ***L. mystacinus*** | ***L. podicipinus*** |
| --- | --- | --- | --- | --- | --- | --- | --- | --- | --- | --- |
| **Alto Paraná Atlantic forests** | 0.00 | 10.96* | 8.44 | 6.45 | 9.89 | 0.00 | 0.49 | 8.29 | 10.64* | 12.25* |
| **Amazon-Orinoco-Southern Caribbean mangroves** | 0.00 | 0.00 | 0.00 | 0.20 | 0.00 | 0.00 | 0.00 | 0.18 | 0.00 | 0.00 |
| **Apure-Villavicencio dry forests** | 0.00 | 0.00 | 0.00 | 0.88 | 0.00 | 0.00 | 0.00 | 0.00 | 0.00 | 0.00 |
| **Araucaria moist forests** | 0.00 | 0.30 | 0.03 | 2.46 | 8.46 | 0.00 | 0.03 | 3.86 | 4.03 | 0.13 |
| **Araya and Paria xeric scrub** | 0.00 | 0.00 | 0.00 | 0.04 | 0.00 | 0.00 | 0.00 | 0.00 | 0.00 | 0.00 |
| **Atlantic Coast restingas** | 0.00 | 0.00 | 0.00 | 0.10 | 0.28 | 0.00 | 0.09 | 0.12 | 0.02 | 0.00 |
| **Atlantic dry forests** | 0.00 | 0.45 | 0.00 | 0.72 | 0.00 | 0.00 | 0.00 | 0.12 | 0.33 | 0.00 |
| **Bahia coastal forests** | 0.00 | 0.02 | 0.00 | 1.40 | 0.00 | 0.00 | 0.00 | 1.73 | 0.51 | 0.00 |
| **Bahia interior forests** | 0.00 | 1.01 | 0.00 | 3.03 | 0.00 | 0.00 | 0.00 | 3.29 | 3.86 | 0.00 |
| **Beni savanna** | 0.00 | 2.33 | 4.87 | 1.67 | 0.00 | 0.00 | 0.00 | 1.97 | 0.59 | 5.74 |
| **Bolivian montane dry forests** | 0.02 | 0.16 | 0.07 | 0.16 | 0.28 | 0.00 | 0.14 | 0.09 | 0.08 | 0.05 |
| **Bolivian Yungas** | 0.00 | 1.16 | 1.57 | 0.96 | 0.00 | 0.00 | 0.01 | 0.29 | 0.03 | 1.78 |
| **Caatinga** | 0.00 | 0.40 | 0.00 | 5.82 | 0.00 | 0.00 | 0.00 | 4.57 | 0.47 | 0.00 |
| **Caatinga Enclaves moist forests** | 0.00 | 0.00 | 0.00 | 0.05 | 0.00 | 0.00 | 0.00 | 0.06 | 0.00 | 0.00 |
| **Campos Rupestres montane savanna** | 0.00 | 0.11 | 0.00 | 0.28 | 0.03 | 0.00 | 0.00 | 0.35 | 0.22 | 0.00 |
| **Caqueta moist forests** | 0.00 | 0.00 | 0.00 | 0.35 | 0.00 | 0.00 | 0.00 | 0.00 | 0.00 | 0.00 |
| **Catatumbo moist forests** | 0.00 | 0.00 | 0.00 | 0.19 | 0.00 | 0.00 | 0.00 | 0.00 | 0.00 | 0.00 |
| **Cauca Valley dry forests** | 0.00 | 0.00 | 0.00 | 0.10 | 0.00 | 0.00 | 0.00 | 0.00 | 0.00 | 0.00 |
| **Cauca Valley montane forests** | 0.00 | 0.00 | 0.00 | 0.26 | 0.00 | 0.00 | 0.00 | 0.00 | 0.00 | 0.00 |
| **Central American dry forests** | 0.00 | 0.00 | 0.00 | 0.00 | 0.00 | 0.00 | 0.00 | 0.00 | 0.00 | 0.00 |
| **Central Andean puna** | 0.00 | 0.06 | 0.00 | 0.02 | 0.11 | 0.00 | 0.08 | 0.08 | 0.08 | 0.00 |
| **Central Andean wet puna** | 0.00 | 0.00 | 0.00 | 0.00 | 0.00 | 0.00 | 0.00 | 0.00 | 0.00 | 0.00 |
| **Cerrado** | 1.53 | 14.39* | 8.07 | 21.51* | 0.21 | 0.00 | 0.02 | 21.17* | 19.64* | 15.84* |
| **Chiquitano dry forests** | 2.11 | 5.16 | 8.10 | 2.74 | 0.00 | 0.10 | 0.00 | 2.39 | 2.68 | 8.11 |
| **Chocó-Darién moist forests** | 0.00 | 0.00 | 0.00 | 0.54 | 0.00 | 0.00 | 0.00 | 0.00 | 0.00 | 0.00 |
| **Cordillera La Costa montane forests** | 0.00 | 0.00 | 0.00 | 0.11 | 0.00 | 0.00 | 0.00 | 0.00 | 0.00 | 0.00 |
| **Cordillera Oriental montane forests** | 0.00 | 0.00 | 0.00 | 0.60 | 0.00 | 0.00 | 0.00 | 0.00 | 0.00 | 0.00 |
| **Dry Chaco** | 70.23* | 25.62* | 30.99* | 6.73 | 10.55* | 98.92* | 33.04* | 11.24* | 18.18* | 12.35* |
| **Eastern Cordillera real montane forests** | 0.00 | 0.00 | 0.00 | 0.01 | 0.00 | 0.00 | 0.00 | 0.00 | 0.00 | 0.00 |
| **Eastern Panamanian montane forests** | 0.00 | 0.00 | 0.00 | 0.04 | 0.00 | 0.00 | 0.00 | 0.00 | 0.00 | 0.00 |
| **Espinal** | 2.08 | 5.25 | 0.25 | 0.08 | 8.36 | 0.00 | 10.78* | 1.72 | 5.68 | 2.80 |
| **Guajira-Barranquilla xeric scrub** | 0.00 | 0.00 | 0.00 | 0.20 | 0.00 | 0.00 | 0.00 | 0.00 | 0.00 | 0.00 |
| **Guianan freshwater swamp forests** | 0.00 | 0.00 | 0.00 | 0.08 | 0.00 | 0.00 | 0.00 | 0.00 | 0.00 | 0.00 |
| **Guianan Highlands moist forests** | 0.00 | 0.00 | 0.00 | 1.15 | 0.00 | 0.00 | 0.00 | 0.03 | 0.00 | 0.00 |
| **Guianan moist forests** | 0.00 | 0.00 | 0.00 | 1.13 | 0.00 | 0.00 | 0.00 | 0.00 | 0.00 | 0.00 |
| **Guianan piedmont and lowland moist forests** | 0.00 | 0.00 | 0.00 | 1.26 | 0.00 | 0.00 | 0.00 | 0.01 | 0.00 | 0.00 |
| **Guianan savanna** | 0.00 | 0.00 | 0.00 | 1.15 | 0.00 | 0.00 | 0.00 | 0.09 | 0.00 | 0.24 |
| **Gurupa varzeß** | 0.00 | 0.00 | 0.00 | 0.11 | 0.00 | 0.00 | 0.00 | 0.17 | 0.00 | 0.28 |
| **High Monte** | 0.13 | 0.01 | 0.00 | 0.00 | 0.01 | 0.00 | 0.00 | 0.69 | 0.04 | 0.00 |
| **Humid Chaco** | 20.26* | 11.03* | 17.62* | 3.68 | 14.61* | 0.78 | 17.43* | 5.30 | 8.27 | 13.04* |
| **Humid Pampas** | 0.05 | 3.02 | 0.00 | 0.00 | 10.22* | 0.00 | 11.51* | 7.26 | 6.08 | 0.34 |
| **Iquitos varzea** | 0.00 | 0.10 | 0.06 | 0.48 | 0.00 | 0.00 | 0.00 | 0.26 | 0.00 | 0.26 |
| **Isthmian-Atlantic moist forests** | 0.00 | 0.00 | 0.00 | 0.09 | 0.00 | 0.00 | 0.00 | 0.00 | 0.00 | 0.00 |
| **Japura-Solimoes-Negro moist forests** | 0.00 | 0.00 | 0.00 | 0.21 | 0.00 | 0.00 | 0.00 | 0.22 | 0.00 | 0.16 |
| **Jurua-Purus moist forests** | 0.00 | 0.00 | 0.00 | 0.38 | 0.00 | 0.00 | 0.00 | 0.19 | 0.00 | 0.03 |
| **La Costa xeric shrublands** | 0.00 | 0.00 | 0.00 | 0.82 | 0.00 | 0.00 | 0.00 | 0.00 | 0.00 | 0.00 |
| **Lara-Falcon dry forests** | 0.00 | 0.00 | 0.00 | 0.02 | 0.00 | 0.00 | 0.00 | 0.00 | 0.00 | 0.00 |
| **Lesser Antillean dry forests** | 0.00 | 0.00 | 0.00 | 0.00 | 0.00 | 0.00 | 0.00 | 0.00 | 0.00 | 0.00 |
| **Llanos** | 0.00 | 0.00 | 0.00 | 4.91 | 0.00 | 0.00 | 0.00 | 0.00 | 0.00 | 0.00 |
| **Low Monte** | 0.03 | 0.00 | 0.00 | 0.00 | 0.00 | 0.00 | 0.00 | 0.11 | 0.01 | 0.00 |
| **Madeira-Tapajós moist forests** | 0.00 | 0.93 | 2.19 | 2.06 | 0.00 | 0.00 | 0.00 | 1.54 | 1.03 | 4.55 |
| **Magdalena Valley dry forests** | 0.00 | 0.00 | 0.00 | 0.26 | 0.00 | 0.00 | 0.00 | 0.00 | 0.00 | 0.00 |
| **Magdalena Valley montane forests** | 0.00 | 0.00 | 0.00 | 0.88 | 0.00 | 0.00 | 0.00 | 0.00 | 0.00 | 0.00 |
| **Magdalena-Urabá moist forests** | 0.00 | 0.00 | 0.00 | 1.02 | 0.00 | 0.00 | 0.00 | 0.00 | 0.00 | 0.00 |
| **Maracaibo dry forests** | 0.00 | 0.00 | 0.00 | 0.10 | 0.00 | 0.00 | 0.00 | 0.00 | 0.00 | 0.00 |
| **Marajó varzea** | 0.00 | 0.00 | 0.00 | 0.45 | 0.00 | 0.00 | 0.00 | 0.06 | 0.00 | 0.30 |
| **Maranhao Babaþu forests** | 0.00 | 0.00 | 0.00 | 0.15 | 0.00 | 0.00 | 0.00 | 1.28 | 0.00 | 0.00 |
| **Mato Grosso seasonal forests** | 0.00 | 0.00 | 0.08 | 2.51 | 0.00 | 0.00 | 0.00 | 0.81 | 0.12 | 0.36 |
| **Mesoamerican Gulf-Caribbean mangroves** | 0.00 | 0.00 | 0.00 | 0.00 | 0.00 | 0.00 | 0.00 | 0.00 | 0.00 | 0.00 |
| **Monte Alegre varzea** | 0.00 | 0.00 | 0.06 | 0.45 | 0.00 | 0.00 | 0.00 | 0.65 | 0.05 | 0.95 |
| **Napo moist forests** | 0.00 | 0.00 | 0.00 | 0.02 | 0.00 | 0.00 | 0.00 | 0.00 | 0.00 | 0.00 |
| **Negro-Branco moist forests** | 0.00 | 0.00 | 0.00 | 0.55 | 0.00 | 0.00 | 0.00 | 0.02 | 0.00 | 0.00 |
| **Northeastern Brazil restingas** | 0.00 | 0.00 | 0.00 | 0.04 | 0.00 | 0.00 | 0.00 | 0.07 | 0.00 | 0.00 |
| **Northern Andean páramo** | 0.00 | 0.00 | 0.00 | 0.00 | 0.00 | 0.00 | 0.00 | 0.00 | 0.00 | 0.00 |
| **Northwestern Andean montane forests** | 0.00 | 0.00 | 0.00 | 0.24 | 0.00 | 0.00 | 0.00 | 0.00 | 0.00 | 0.00 |
| **Orinoco Delta swamp forests** | 0.00 | 0.00 | 0.00 | 0.06 | 0.00 | 0.00 | 0.00 | 0.00 | 0.00 | 0.00 |
| **Orinoco wetlands** | 0.00 | 0.00 | 0.00 | 0.03 | 0.00 | 0.00 | 0.00 | 0.00 | 0.00 | 0.00 |
| **Panamanian dry forests** | 0.00 | 0.00 | 0.00 | 0.00 | 0.00 | 0.00 | 0.00 | 0.00 | 0.00 | 0.00 |
| **Pantanal** | 1.16 | 5.14 | 8.49 | 2.16 | 0.00 | 0.00 | 0.00 | 2.59 | 0.70 | 7.33 |
| **Pantepui** | 0.00 | 0.00 | 0.00 | 0.33 | 0.00 | 0.00 | 0.00 | 0.00 | 0.00 | 0.00 |
| **Paraguana xeric scrub** | 0.00 | 0.00 | 0.00 | 0.00 | 0.00 | 0.00 | 0.00 | 0.00 | 0.00 | 0.00 |
| **Paraná flooded savanna** | 0.50 | 1.36 | 0.43 | 0.17 | 2.43 | 0.00 | 2.86 | 0.68 | 0.90 | 1.04 |
| **Patya Valley dry forests** | 0.00 | 0.00 | 0.00 | 0.00 | 0.00 | 0.00 | 0.00 | 0.00 | 0.00 | 0.00 |
| **Pernambuco coastal forests** | 0.00 | 0.00 | 0.00 | 0.23 | 0.00 | 0.00 | 0.00 | 0.32 | 0.00 | 0.00 |
| **Pernambuco interior forests** | 0.00 | 0.00 | 0.00 | 0.26 | 0.00 | 0.00 | 0.00 | 0.35 | 0.00 | 0.00 |
| **Peruvian Yungas** | 0.00 | 0.00 | 0.00 | 0.30 | 0.00 | 0.00 | 0.00 | 0.00 | 0.00 | 0.01 |
| **Purus varzea** | 0.00 | 0.00 | 0.00 | 0.49 | 0.00 | 0.00 | 0.00 | 0.36 | 0.00 | 0.38 |
| **Purus-Madeira moist forests** | 0.00 | 0.00 | 0.00 | 0.21 | 0.00 | 0.00 | 0.00 | 0.40 | 0.01 | 0.40 |
| **Rio Negro campinarana** | 0.00 | 0.00 | 0.00 | 0.40 | 0.00 | 0.00 | 0.00 | 0.09 | 0.00 | 0.00 |
| **Santa Marta montane forests** | 0.00 | 0.00 | 0.00 | 0.04 | 0.00 | 0.00 | 0.00 | 0.00 | 0.00 | 0.00 |
| **Serra do Mar coastal forests** | 0.00 | 0.46 | 0.00 | 1.37 | 3.69 | 0.00 | 0.00 | 1.88 | 2.25 | 0.00 |
| **Sin· Valley dry forests** | 0.00 | 0.00 | 0.00 | 0.33 | 0.00 | 0.00 | 0.00 | 0.00 | 0.00 | 0.00 |
| **Solimoes-Japura moist forests** | 0.00 | 0.00 | 0.00 | 0.02 | 0.00 | 0.00 | 0.00 | 0.00 | 0.00 | 0.00 |
| **South American Pacific mangroves** | 0.00 | 0.00 | 0.00 | 0.05 | 0.00 | 0.00 | 0.00 | 0.00 | 0.00 | 0.00 |
| **Southern Andean steppe** | 0.00 | 0.00 | 0.00 | 0.00 | 0.00 | 0.00 | 0.00 | 0.13 | 0.00 | 0.00 |
| **Southern Andean Yungas** | 1.50 | 2.01 | 1.14 | 0.69 | 3.70 | 0.19 | 3.29 | 0.96 | 1.45 | 0.08 |
| **Southern Atlantic mangroves** | 0.00 | 0.00 | 0.00 | 0.11 | 0.18 | 0.00 | 0.00 | 0.17 | 0.06 | 0.00 |
| **Southern Cone Mesopotamian savanna** | 0.36 | 1.03 | 1.69 | 0.36 | 2.09 | 0.00 | 1.17 | 0.49 | 0.83 | 1.25 |
| **Southwest Amazon moist forests** | 0.02 | 3.26 | 5.52 | 4.46 | 0.00 | 0.00 | 0.00 | 3.30 | 0.73 | 7.73 |
| **Tapajós-Xingu moist forests** | 0.00 | 0.00 | 0.00 | 0.19 | 0.00 | 0.00 | 0.00 | 0.20 | 0.00 | 0.11 |
| **Tocantins/Pindare moist forests** | 0.00 | 0.00 | 0.00 | 0.29 | 0.00 | 0.00 | 0.00 | 0.28 | 0.00 | 0.00 |
| **Trinidad and Tobago moist forests** | 0.00 | 0.00 | 0.00 | 0.06 | 0.00 | 0.00 | 0.00 | 0.00 | 0.00 | 0.00 |
| **Uatuma-Trombetas moist forests** | 0.00 | 0.00 | 0.00 | 1.26 | 0.00 | 0.00 | 0.00 | 0.75 | 0.00 | 1.21 |
| **Ucayali moist forests** | 0.00 | 0.00 | 0.00 | 0.04 | 0.00 | 0.00 | 0.00 | 0.00 | 0.00 | 0.00 |
| **Uruguayan savanna** | 0.00 | 4.27 | 0.34 | 2.87 | 24.86* | 0.00 | 19.05* | 6.37 | 10.29* | 0.83 |
| **Venezuelan Andes montane forests** | 0.00 | 0.00 | 0.00 | 0.15 | 0.00 | 0.00 | 0.00 | 0.00 | 0.00 | 0.00 |
| **Western Ecuador moist forests** | 0.00 | 0.00 | 0.00 | 0.01 | 0.00 | 0.00 | 0.00 | 0.00 | 0.00 | 0.00 |
| **Xingu-Tocantins-Araguaia moist forests** | 0.00 | 0.00 | 0.00 | 1.12 | 0.00 | 0.00 | 0.00 | 0.40 | 0.11 | 0.06 |
| **Total** | 100.00 | 100.00 | 100.00 | 100.00 | 100.00 | 100.00 | 100.00 | 100.00 | 100.00 | 100.00 |
